# Network-based analysis reveals genetic diversity of human-brain expressed genes with high evolutionary rates

**DOI:** 10.3389/fnins.2026.1866277

**Published:** 2026-10-20

**Authors:** Yuequn Ma, Jupeng Yuan, Ju Wang

**Affiliations:** 1 Shandong Provincial Key Laboratory of Precision Oncology, Shandong Cancer Hospital and Institute, Shandong First Medical University, Shandong Academy of Medical Sciences, Jinan, Shandong, China; 2 School of Biomedical Engineering, Tianjin Medical University, Tianjin, China; 3 Department of Radiation Oncology, The First Hospital of China Medical University, Shenyang, Liaoning, China

**Keywords:** enrichment analysis, gene evolution, nervous system, protein-protein interaction network, single cell analysis

## Abstract

**Introduction:**

Comparing the gene expression profiles of human and non-human primate brains can provide essential insights to understand the structural and functional disparities between these species, as well as the origins of many diseases. In recent years, the availability of genomic and transcriptomic data of the human brain has made it feasible to explore the evolutionary features of the brain-expressed genes at the molecular level on a large scale. Analyses of human and non-human primate brain cell-type-specific expressed genes with elevated evolutionary rates can uncover the genetic and functional diversity intrinsic to the human brain.

**Methods:**

In this study, we try to explore the genetic diversity of the human brain by analyzing genes with divergent evolutionary rates in human and macaque brains, as well as in the human lineage.

**Results:**

We identified 450 genes with high evolutionary rates between human and macaque, and 534 genes between human populations. Topological characteristics showed that these genes had lower protein-protein interaction network node degree. We also found that pathways related to neurological diseases, immune, metabolism, and signaling transduction were enriched in the genes of high evolutionary rates between human and macaque. We further identified 97 and 137 potential genes with differential evolution features between and within species.

**Discussion:**

Our results reveal the genetic diversity of evolutionarily variable specific-expressed genes in the human brain and provide complementary findings for human brain evolution at the transcriptional level.

## Introduction

The human brain is distinguished from other primate brains by its large volume, intricate organizational structure, and advanced cognitive capacities. Like other organs, the human brain is also the product of millions of years of evolution. In the process of evolution, the brain has continuously been reshaped in both size and structure to adapt to changes in the environment and climate, progressively optimizing its capacity to execute sophisticated cognitive functions (Lindhout et al., 2024; Sousa et al., 2017a; Vallender et al., 2008; Zhou et al., 2024). Thus, comparative analysis of the brain of humans and other species within the context of evolution can provide important insights into the neuroanatomical and molecular changes associated with the distinctive cognitive abilities of humans (Zhu et al., 2018).

Over the past years, substantial progress has been made in characterizing the anatomical and physiological substrates of human and other primate brains, greatly advancing our understanding of the unique structural, cellular, and neuronal characteristics of the human brain (Sakai et al., 2012; Vallender et al., 2008). Especially, recent technological innovations make it easier to obtain larger brain tissue datasets and perform more accurate measurements, which enable researchers to explore the unique features of the human nervous system across multiple biological levels (Pollen et al., 2019; Sousa et al., 2017b; Zhu et al., 2018). For instance, an earlier transcriptomic analysis revealed that the human brain may have a distinctive pattern of gene expression relative to non-human primates, with over 100 genes exhibiting elevated expression in the human cerebral cortex (Cáceres et al., 2003). A recent study via transcriptome sequencing found regional and cell-type-specific gene expression patterns in 16 brain regions of humans, chimpanzees, and macaques (Sousa et al., 2017b). Using imaging technology comparisons between human and chimpanzee brains, special evolutionary characteristics of brain circuits have been identified (van den Heuvel et al., 2019). Neuroimaging and genetic analysis of the cerebral cortex of humans and non-human primates showed that the relationship between ventricular volume and variations in the ASPM (Assembly Factor For Spindle Microtubules) gene contributes substantially to structural brain evolution (Singh S. V. et al., 2019). It has further revealed that the evolution of the human genome is involved in the expansion and cortical organization of cognitive functional networks in the brain (Singh S. V. et al., 2019; Wei et al., 2019). Collectively, existing studies have demonstrated the diverse molecular and cellular features underlying the unique evolutionary traits of the human brain. Previous studies have mapped gene dN/dS values to the human brain and revealed the evolutionary rules of human neurogenetics (Kaczanowska et al., 2022). And compared the evolutionary characteristics of related genes across humans, primates, and species with distinct brain sizes (Ogawa and Vallender, 2014). However, due to the extreme structural complexity of the human brain, current academic understanding regarding the functional characteristics and genetic diversity of brain evolutionary genes, as well as the mining of potential evolutionary genes based on PPI networks, remains limited.

To date, numerous genes related to the evolution of the structure and functional specificities of the human brain have been identified (Fiddes et al., 2018; Vallender et al., 2008). Under the pressure of natural selection, the genes related to the same phenotypic trait tend to be affected as a whole because mutations in a single gene can induce alterations in its co-expressed genes or networks (Daub et al., 2015). Recent studies have used evolutionary analytical methods to determine the gene sets under selective pressure, or to explore the evolutionary patterns of individual genes across human populations, and promising results have been obtained (Cagliani et al., 2014; Stobdan et al., 2017). The classic framework for detecting selection pressure on coding sequences is the McDonald-Kreitman test, which integrates interspecies divergence and intraspecies polymorphism data to test the neutral evolution hypothesis.^1^ Following this analytical strategy, our previous study performed pathway-level selection pressure analyses on human brain-expressed genes using dN/dS and pN/pS, and identified a set of functional pathways potentially subject to lineage-specific divergence (Ma et al., 2021). Nevertheless, such aggregated pathway-level analyses pool evolutionary metrics for all genes within a pathway and cannot distinguish whether pathway-associated evolutionary signals stem from the pathway as a whole or from a small subset of evolutionarily anomalous individual genes inside the pathway. Therefore, systematic network-based analysis of the genes related to the evolutionary rates of the human brain is necessary. Such approaches enable comprehensive exploration of the genetic diversity of genes expressed in the human brain, rather than relying merely on individual gene analyses.

This study attempts to investigate the biological functions of genes with high evolutionary rates and identify potential evolutionary genes associated with cell-type-specific expression in the human brain, through comparisons with the macaque. A species with a nervous system similar to that of humans and serves as one of the most widely used experimental models in phylogeny analysis (Cagliani et al., 2014; Sousa et al., 2017a; Zhu et al., 2018). Then, based on the protein-protein interaction (PPI) network, the topological characteristics and functions of genes that distinguish humans from macaques or between subpopulations were explored. We also predicted the specific protein networks of evolutionarily conserved genes using these evolution-related genes. This study revealed the genetic diversity of evolutionarily variable, specifically expressed genes in the human brain and provided important complementary findings for understanding human brain evolution at the transcriptional level.

## Materials and methods

### Genes expressed in the nervous system of humans and macaques

Gene expression datasets from human and macaque brains were retrieved from published transcriptomic resources (Zhu et al., 2018). In the study, gene expression patterns in brain tissue or single cells from post-mortem human and macaque specimens were analyzed by RNA sequencing (RNA-seq). Using the quality-controlled specificity-score matrix, we retained genes with specificity scores > 0 from adult human and macaque brains in the present study, resulting in 23,467 human brain genes and 17,102 macaque brain genes (Supplementary Table 18).

### dN/dS between human and macaque genes and pN/pS in human genes

At the molecular level, evolutionary constraint on a gene is often estimated by comparing the number of non-synonymous substitutions per non-synonymous site (dN) and the number of synonymous substitutions per synonymous site (dS) between homologous gene sequences (Yang and Nielsen, 2002). The protein-coding genes of human and macaque were retrieved from Ensembl99^2^ on 5 February 2020 (Yates et al., 2020). Only the homologous genes with synonymous (dS) and non-synonymous (dN) values and the orthologous genes with dS not equal to zero were retained. Then dN/dS ratios were used to measure the strength of natural selection on homologous genes in the human and macaque genomes. Altogether, 18,409 genes with valid dN/dS ratios were obtained (Supplementary Table 1).

Similarly, pN/pS (the ratio of non-synonymous to synonymous in different populations of the same species) is often used to measure the selection pressure on genes in the same genome. In this study, the pN/pS ratios for human genes were estimated based on the variation data from the 1,000 Genomes Project^3^ (Sudmant et al., 2015), which included information on 84.4 million variants in 2,504 individuals from 26 populations. ANNOVAR was used to identify mutation sites in the human genome (Wang et al., 2010), and 376,047 synonymous mutation sites and 594,411 non-synonymous mutation sites in exons were obtained. Eventually, 18,833 genes with pN/pS ratios were kept (Supplementary Table 2). All variants from the 1000 Genomes Project were retained without minor allele frequency filtering, incorporating both common and rare variants. Genes with zero synonymous variants were excluded, whereas no additional hard cutoff was imposed on the total number of synonymous variants.

To allow direct and uniform comparison for the evolutionary characteristics of brain-associated genes among different species and within a given species (Supplementary Figure 7), the three lists of gene, i.e., genes expressed in the brain of human and macaque, genes with dN/dS ratios, and genes with pN/pS ratios, were compared and 13,522 genes with both dN/dS and pN/pS ratios and were expressed in human and macaque brain were collected (Supplementary Table 3). We extracted CDS length and GC content, evaluated their correlations with dN/dS and pN/pS via Spearman’s rank correlation test, and defined dS-saturated genes (Supplementary Table 20).

### Correlation between evolutionary-rate metrics and cell-type specificity scores

To explore the associations between evolutionary-rate metrics and cell-type expression divergence reported in the original dataset, we retained all genes with specificity scores > 0 across human and macaque brain regions. The maximal cell-type specificity score per gene across all cell-types was computed for subsequent statistical analysis. Spearman’s rank-correlation coefficient was applied to evaluate correlations between dN/dS, pN/pS and maximal specificity scores. For group-wise comparison of specificity-score distributions, the Kruskal-Wallis test was performed for overall multi-group comparison, followed by pairwise Wilcoxon rank-sum tests with multiple-test correction.

### Genes under differential evolutionary constraints in the brain of human and macaque

As mentioned above, the dN/dS ratio can be used to measure the evolutionary constraint on a gene in two or more species, and a gene with a dN/dS ratio significantly different from the majority of genes in the species compared may be under specific selection pressure in evolution and have different evolutionary features. Similarly, a gene with a pN/pS ratio significantly different from the majority of genes in the human genome may be under specific evolutionary constraints in the development of the human lineage. Thus, we further identified the genes with significantly larger/smaller dN/dS or pN/pS ratios. The interquartile range (IQR) rule, a method widely used to detect outliers in biological or medical samples, as shown in Equation 1 has been adopted in a wealth of molecular evolutionary studies and has consistently adopted the 1.5 × IQR cutoff to screen genes with aberrant evolutionary rates (Jeong et al., 2022; Long et al., 2016). We adopted it to detect genes with outlying dN/dS or pN/pS ratios.

IQR could be calculated by:


$$\mathrm{IQR}=Q_{3}-Q_{1}$$ (1)

with Q1 being the first quartile or the 25th percentile of the data, and Q3 the third quartile) or the 75th percentile of the data.

Then, the upper boundary and lower boundary were defined as:


$$u=Q_{3}+1.5\times \mathrm{IQR}$$ (2)

$$l=Q_{1}+1.5\times \mathrm{IQR}$$ (3)

Then, genes having dN/dS ratios larger than Equation 2 or smaller than Equation 3 were selected, which were defined as genes under differential evolutionary constraints between species. Those with pN/pS ratios larger than Equation 2 or smaller than Equation 3 were defined as genes under differential evolutionary constraints within species. Notably, the IQR of dN/dS was 0.32 [Q_3_ (0.4)−Q_1_ (0.08)], and the IQR of pN/pS was 1.132 [Q_3_ (2.25)−Q_1_ (1.118)]; Equation 3 was negative. Since both dN/dS and pN/pS in this study were positive, there were no lower-side outliers, and only upper-side outliers were filtered. Accordingly, only genes with greater than Equation 2 were retained for subsequent analyses in this study. In this study, genes with dN/dS ratios exceeding Equation 2 were defined as DEinter genes, while those with pN/pS ratios greater than Equation 2 were defined as DEintra genes.

### Functional enrichment analysis on genes under high evolutionary constraints

The Database for Annotation, Visualization and Integrated Discovery (DAVID) (Huang da et al., 2009) was utilized to explore the biological themes enriched in the DEinter genes and DEintra genes. DAVID is an online tool designed to identify the biological functions of genes or proteins, including GO, KEGG, REACTOME, and other pathway databases. Each database has a special way to define pathways, and different databases often have pathways with the same or similar names that may comprise different genes. In this study, only the enrichment analysis results with FDR < 0.2 in the KEGG database were retained; GO BP terms were complemented.

### Network degree distribution of genes under high evolutionary constraints

The network topological properties of the DEinter genes and DEintra genes were then explored in the human PPI network. First, we integrated data from six common PPI databases from Protein Interaction Network Analysis, namely BioGRID, IntAct, DIP, MINT, MIPS/MPact, and HPRD (Cowley et al., 2012), which was further merged with a previously established human protein interaction network (Menche et al., 2015). Next, we removed redundant pairs and PPI pairs from the Negatome database. The Negatome database contains manually annotated PPI pairs that do not have direct physical interaction (Smialowski et al., 2010). Finally, we obtained a PPI network with 16,022 nodes (genes/proteins) and 228,070 interaction edges of protein products.

In a PPI network, the degree of a node is the number of nodes directly connected with it, and a node with a high degree is considered to be critical to the structure or characteristics of the entire biological network (Mondragón, 2020). We mapped the DEinter genes and DEintra genes onto the PPI network and calculated their degrees.

### Specific subnetwork for genes under high evolutionary constraints

We then constructed networks for DEinter genes and DEintra genes based on the human PPI network via the Steiner minimal tree algorithm (Klein and Ravi, 1995). Steiner minimal tree algorithm uses a greedy search strategy to build a subnetwork that connects the most input nodes with the least number of interconnected nodes. We extracted two subnetworks from the PPI network using the DEinter genes or DEintra genes as the input nodes. For comparison purposes, we generated 1,000 simulated subnetworks serving as the null hypothesis, each matching the DEinter and DEintra subnetworks in node and edge counts (Erdos and Rényi, 1960). Then, permutation tests were used to assess the significance of deviation of actual values from a random distribution. The tool GenRev (Zheng and Zhao, 2012) was used to build the subnetwork for DEinter genes and DEintra genes, and the Erdős-Rényi model was adopted to construct the simulated subnetworks. We calculated global clustering coefficient and average shortest-path length for 1,000 Erdős-Rényi simulated networks to build the null distribution. Permutation tests revealed real subnetworks had higher clustering coefficients and shorter paths (*P*<0.001), supporting their BA scale-free architecture. Vertex and edge betweenness centrality were calculated via igraph (v2.3.2). After outlier removal, we compared networks filtered by node degree and betweenness centrality.

### Single-cell validation

We integrated human and macaque single-cell brain datasets from the GEO database (GSE293664, GSE302350, GSE319557, and GSE183052). The obtained gene expression matrices were independently processed in R (v4.5.0) with the Seurat package (v5.0.0). The expression matrix was normalized via LogNormalize, and variance stabilizing transformation (vst) was applied to screen 2,000 highly variable genes. Principal component analysis (PCA) was performed based on the normalized highly variable genes, and the top 30 principal components were extracted for subsequent dimensionality reduction analysis. To eliminate technical batch effects across the four independent datasets, Harmony batch correction was implemented on the PCA outputs. After correction, UMAP dimensionality reduction was conducted using the top 30 Harmony-corrected vectors to construct a cell neighborhood network, followed by unsupervised cell clustering. All other analyses were performed using default parameters of the Seurat package. The DEinter gene set and enriched biological processes were validated by GSVA (v2.4.0) and clusterProfiler (v4.18.4) analyses, respectively.

## Results

### Network properties of genes under high evolutionary constraints in brain

Among the 13,522 genes expressed in human and macaque brains with qualified dN/dS and pN/pS ratios, 450 DEinter genes with significantly larger dN/dS ratios relative to most brain-expressed genes in humans and macaques were identified (Supplementary Table 4). Meanwhile, 534 DEintra genes with significantly larger pN/pS ratios relative to most brain-expressed genes in humans were identified (Supplementary Table 5). Genes showing cell-type-specific expression across distinct brain cell types tend to have higher dN/dS or pN/pS ratios. Compared with the majority of genomic genes, these genes exhibit markedly elevated interspecies or intraspecies evolutionary rates. For example, defensin beta (DEFB) possibly is involved in the proliferation of defensins associated with coevolution; Rh blood group CcEe antigens (RHCE) have replication events in common ancestors and mutate in the evolution of different species; apolipoprotein C2 (APOC2) diverges among different populations (Barrán-Berdón et al., 2013; Radhakrishnan et al., 2005). DEFB exhibits genetic diversity both across species and within human populations.

The topological properties of the DEinter and DEintra genes in the human PPI network were analyzed to explore their biological features. Of the 450 DEinter genes, 323 could be mapped to the PPI network, and the median degree was 5. Of the 534 DEintra genes, 415 could be mapped to the PPI network, and the median degree was 6. For comparison, we also calculated the degree of all the brain-expressed genes with dN/dS and pN/pS ratios. Of the 13,522 genes, 11,712 could be mapped to the PPI network, and the median degree was 10 (Figure 1). Node degrees ranged broadly from 1 to over 500, and the degree distributions were right-skewed, with most genes maintaining only a few connections with other genes, and a minority of genes had a large number of connections. Compared with the DEinter genes, the median degree of DEintra genes was closer to that of genes expressed in the brain of humans and macaques. Power-law distribution analysis and Vuong likelihood ratio test revealed a two-sided *P*-value of 0.0919 for the power-law decay distributions between DEinter and DEintra genes, with distinct slopes observed in the log-log plots (Supplementary Figure 2). Nevertheless, the two-sided *P*-values for comparisons between DEinter and brain genes, as well as between DEintra and brain gene sets, were greater than 0.5. Hub genes with a high degree are more likely to trigger system collapse upon targeted knockout, rendering them highly conserved over evolution. This represents a well-established genome-wide trend in molecular evolution and is not restricted to brain-expressed genes. In contrast, regulatory nodes with low degree predominantly govern specialized functional subsystems such as cell differentiation. Random loss of such peripheral nodes barely impairs the global connectivity of the network, suggesting that these nodes may undergo weaker purifying selection and tend to evolve at relatively faster evolutionary rates (Wolf et al., 2021). Both DEinter and DEintra genes contain numerous low-degree peripheral genes in the human PPI network. Many of the genes with lower degrees are involved in biological processes such as signal transduction, the nervous system, immune function, and metabolism. For example, protein tyrosine phosphatase receptor type C (PTPRC) has positive selection among species, and the CD52 molecule (CD52) carries functional variants, which may alter GPI-anchor formation efficiency (Oko et al., 2009; Ortiz et al., 2009). Evolutionary analysis has revealed differences in immune genes (eg, pyrin and HIN domain family member 1 (PYHIN1), absent in melanoma 2 (AYM2), glycophorin A (GYPA), B and T lymphocyte associated (BTLA), and differences in diet between species leading to peroxisomal trans-2-enoyl-CoA reductase (PECR) specific metabolism (Ko et al., 2011; Moser et al., 2013; Partyka et al., 2015). GYPA exhibits genetic diversity both across species and within human populations. CD3E and GZMB are canonical immune cell markers that execute immunomodulatory functions within the nervous system. The expression patterns of these DEinter and DEintra genes reflect genetic polymorphisms of transcribed genes in the human brain that regulate nervous system function and exert direct or indirect effects on neural activity. The results are consistent with previous studies (Berghoff et al., 2021; Ireland et al., 2017). We found that both DEinter and DEintra genes exhibited lower median node degrees compared with the brain-expressed background genes. Genes with lower node degrees act as peripheral nodes during species evolution and experience weaker purifying selection, rendering them more prone to genetic diversification. Therefore, investigation of brain-specific expressed genes with high evolutionary rates helps elucidate the genetic diversity of transcribed genes in the human brain.

**FIGURE 1:**
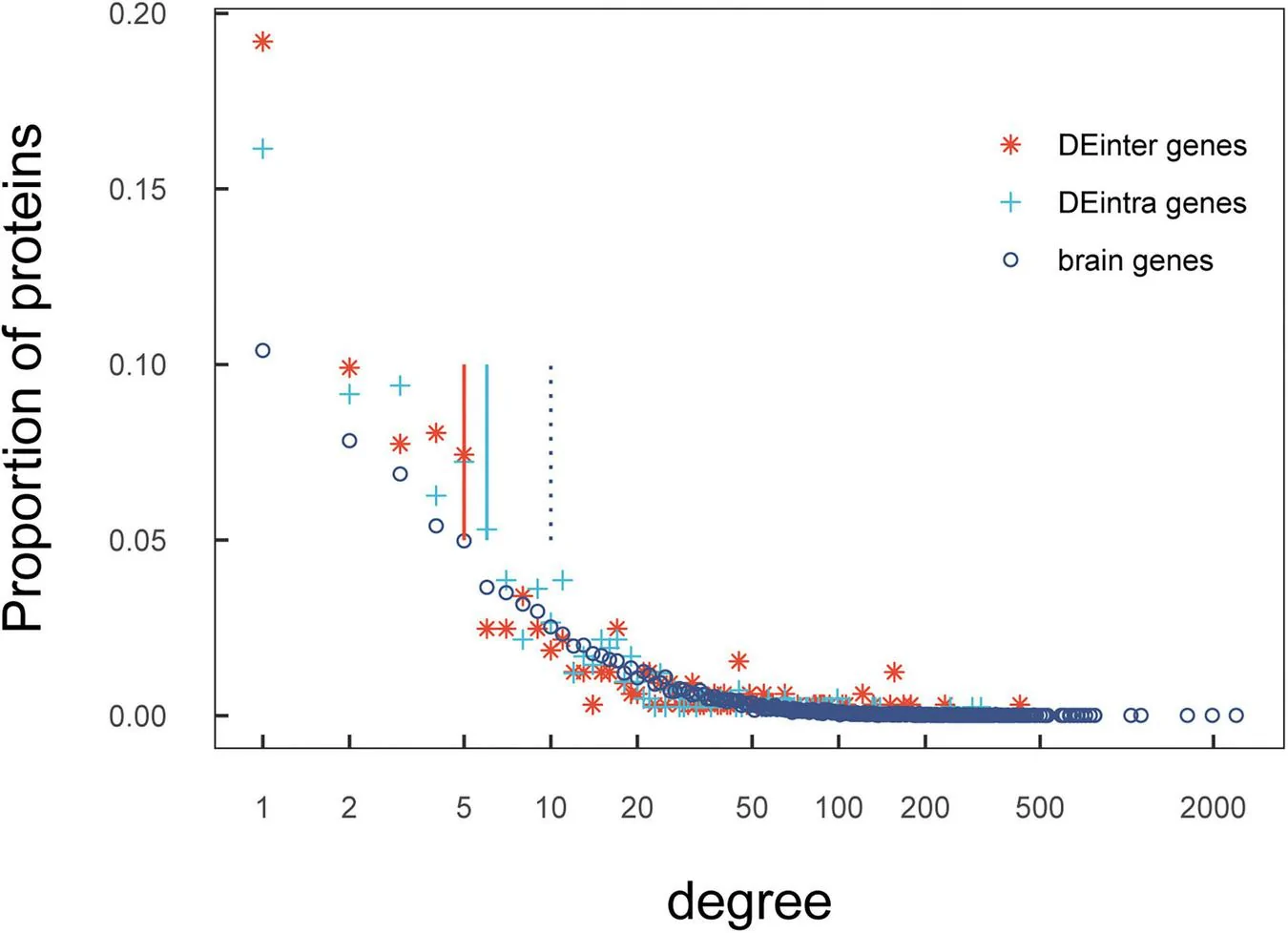
Distribution of degree for genes in DEinter and DEintra, i.e., genes with outlying dN/dS or pN/pS ratios, as well as those expressed in human brain. Vertical line denotes the mean degree. Red line represents DEinter genes, blue line represents DEintra genes, and deep blue line represents brain genes.

To explore the correlations between evolutionary-rate metrics and cell-type expression divergence reported in the original dataset, we performed correlation analyses. Our results showed that both interspecific dN/dS and intraspecific pN/pS were negatively correlated with cell-type specificity scores, meaning that genes with higher cell-type specificity tended to evolve more slowly (Supplementary Figures 8a–c). The cell-type specificity scores of DEinter and DEintra genes showed significant differences relative to the background gene set, whereas no statistically significant difference was found between DEinter and DEintra groups (Supplementary Figures 8d,e). CDS length and GC content showed significant weak negative correlations with dN/dS and pN/pS, and dS-saturated genes generally exhibited lower evolutionary rates (Supplementary Figures 9a–c).

### Function enrichment on genes under high evolutionary constraints in the brain

There were 13 pathways significantly enriched in DEinter genes (Table 1), which were pathways under high evolutionary constraints in the evolution of humans and macaques. These pathways were mainly related to signal transduction, neurodegenerative diseases, and immune function. Some pathways were related to neurological diseases, such as Alzheimer’s disease (AD), Parkinson’s disease (PD), and Huntington’s disease (HD). These findings reveal interspecific evolutionary divergence in functional pathways of brain-expressed genes and are consistent with experimental results of AD and PD differences between species (Cruciani et al., 2024). We also identified the signaling pathways (cytokine-cytokine receptor interaction, cell adhesion molecules), metabolic pathways (oxidative phosphorylation, non-alcoholic fatty liver disease), and six immune pathways, including three immune system-related, and three infectious disease-related pathways. Gene expression analysis indicated that interspecies differences existed in oxidative stress and cytokine levels (Zhu et al., 2018). Enrichment results revealed that DEinter genes were closely involved in pathways associated with neurological disorders, immunity, and infectious diseases, suggesting extensive functional genetic diversity of brain-specific expressed genes across species. In addition, several GO terms related to taste and smell stimuli were enriched in DEinter genes, indicating pervasive genetic polymorphism of sensory system pathways across species (Supplementary Table 6).

**TABLE 1:** Pathways enriched in genes of DEinter.

Pathway	P-value	FDR^a^	Gene included
Measles	8.60 × 10^–5^	0.012	MAVS, IL13, EIF2AK2, CD3G, TNFRSF10A, CD46, CD3E, FCGR2B, TLR4, BBC3, IFNAR1
Cytokine-cytokine receptor interaction	2.50 × 10^–4^	0.017	CCL11, CCL22, CCL20, IL24, IL13, IL18, IL19, TNFRSF10A, EDA2R, CXCL16, GHR, CCL1, CCL26, IFNAR1
Alzheimer’s disease	2.30 × 10^–3^	0.100	APH1A, COX7B, NDUFA5, UQCRQ, NDUFA4, NDUFB4, NDUFS2, NDUFC2, COX7A2, SNCA
Parkinson’s disease	2.90 × 10^–3^	0.100	COX7B, NDUFA5, UQCRQ, NDUFA4, NDUFB4, NDUFS2, NDUFC2, COX7A2, SNCA
Hematopoietic cell lineage	3.70 × 10^–3^	0.100	GYPA, CD1E, CD3G, CD59, CD3E, CD34, CD55
Non-alcoholic fatty liver disease (NAFLD)	4.30 × 10^–3^	0.100	COX7B, XBP1, NDUFA5, UQCRQ, NDUFA4, NDUFB4, NDUFS2, NDUFC2, COX7A2
Huntington’s disease	5.50 × 10^–3^	0.110	COX7B, NDUFA5, UQCRQ, NDUFA4, NDUFB4, NDUFS2, TFAM, NDUFC2, COX7A2, BBC3
Oxidative phosphorylation	7.70 × 10^–3^	0.130	COX7B, NDUFA5, UQCRQ, NDUFA4, NDUFB4, NDUFS2, NDUFC2, COX7A2
Malaria	9.60 × 10^–3^	0.140	GYPA, VCAM1, KLRB1, IL18, TLR4
Influenza A	9.80 × 10^–3^	0.140	IL33, MAVS, RNASEL, IL18, EIF2AK2, TNFRSF10A, NXT2, TLR4, IFNAR1
Cell adhesion molecules (CAMs)	1.10 × 10^–2^	0.140	CD274, VCAM1, SELPLG, PTPRC, CD226, CD58, TIGIT, CD34
NOD-like receptor signaling pathway	1.50 × 10^–2^	0.180	CASP5, CARD8, CARD6, IL18, NLRP1
Natural killer cell mediated cytotoxicity	1.80 × 10^–2^	0.190	RAET1E, TYROBP, GZMB, CD48, TNFRSF10A, CD244, IFNAR1

^a^Pathways with FDR < 0.2 are listed.

For the DEintra genes, no enriched pathway was detected, yet several enriched GO biological process terms were retrieved. The terms mainly included immune response, cellular processes, energy metabolism, and genetic information processes. These terms exhibit widespread genetic polymorphism within human populations, which may be associated with living environments; for example, malaria in Africa was only prevalent in a limited space, and people at high altitude have specific immune mechanisms (Baye et al., 2009; de Filippo et al., 2016; Partyka et al., 2015). Particularly, biological processes such as “responds to auditory stimulus” were enriched, indicating pervasive genetic polymorphism among distinct human populations. Unlike interspecies GO signatures, intraspecific DEintra gene enrichments did not feature taste and olfactory sensory terms (Supplementary Table 7). Nevertheless, whether these significantly enriched pathways are upregulated or downregulated in the human brain remains unclear.

### Specific subnetwork for genes under high evolutionary constraints

To further explore the potential genes with specific evolutionary rates at interspecific and intraspecific levels, we constructed two subnetworks for the genes in DEinter and DEintra based on the Steiner minimal tree algorithm. The two subnetworks contained 397 nodes and 883 edges (interactions), and 534 nodes and 1,136 edges, respectively (Figure 2). In the subnetwork corresponding to the DEinter set, 397 nodes and 883 edges (interactions between genes/proteins) were included. Permutation test results showed that the global clustering coefficients of the real subnetworks were significantly higher than those of simulated networks, and the average shortest path lengths were significantly shorter (permutation test *P* < 0.001), indicating that both real subnetworks conformed to the BA scale-free network model. We also calculated betweenness centrality and verified that the overlap ratios of nodes and edges between betweenness centrality networks and the two real networks were all above 98.8% (Supplementary Figure 1). Considering the complexity of the DEinter and DEintra subnetworks, we extracted clustered subnetworks separately using MCODE (v2.0.3) (Supplementary Figure 6). Among the genes in this subnetwork, 300 were from the DEinter genes, which accounted for 75.6% of the genes (397 altogether) in the subnetwork, indicating a high coverage rate. The subnetwork of DEintra genes consisted of 534 nodes and 1,136 edges. Among the genes in this subnetwork, 397 genes from the DEintra set, accounting for 74.3% of the genes (534 altogether) in the subnetwork. A total of 97 and 137 genes (linker genes) were outside the DEinter and DEintra sets, respectively. These linker genes were necessary to build the subnetworks, as each interacted with two or more genes in the original gene sets. Given their tight linkage to DEinter or DEintra genes, these linker genes may serve as bridges for signal transmission and functional regulation within the gene interaction network (Helsen et al., 2019).

**FIGURE 2:**
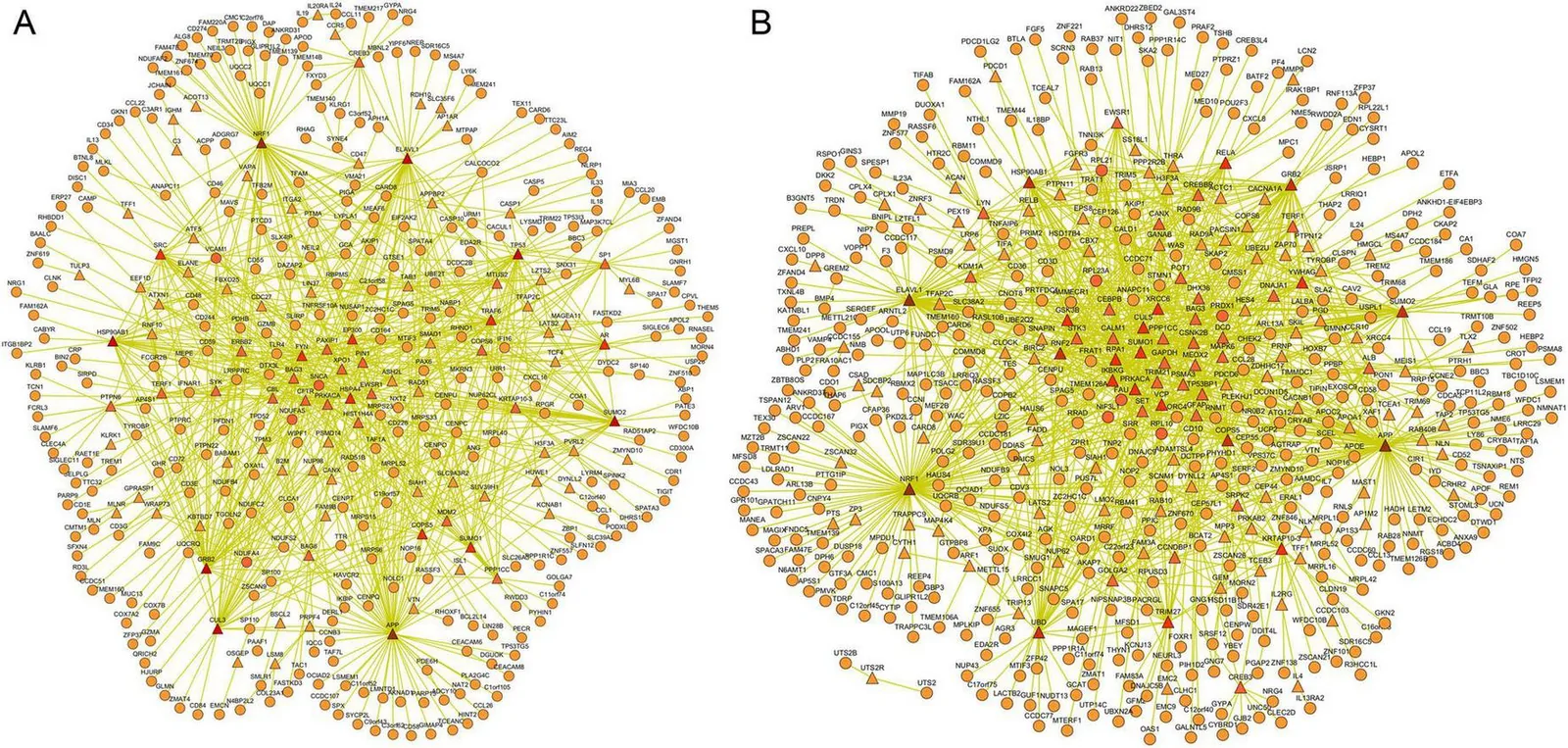
Standing variation gene-specific subnetwork. **(A)** Specific subnetwork for genes with differential standing variation between human and macaque, which consists of 397 nodes and 883 edges. **(B)** Specific subnetwork for genes with differential standing variation in the human lineage, which consists of 534 nodes and 1,136 edges. Circular vertices indicate outliers; triangle vertices indicate potential genes. The color scale of vertices corresponds to the degree value of each node. Yellow edges indicate direct physical PPI between two genes/proteins.

Among the 97 genes, existing literature has demonstrated that some genes exhibit interspecies features including differential brain expression and transcriptional regulatory divergence, reflecting widespread sequence and regulatory polymorphism of brain-specific expressed genes across species, e.g., SRC proto-oncogene, non-receptor tyrosine kinase (SRC), E1A binding protein p300 (EP300), and C-C motif chemokine receptor 5 (CCR5) displays sequence and transcriptional regulatory divergence across primates, which supports its status as a candidate gene with interspecific and intraspecific evolutionary traits (Lindskog et al., 2014; Mummidi et al., 2000; Picard et al., 2002). Particularly, eukaryotic translation elongation factor 1 delta (EEF1D) has been demonstrated to be differentially expressed in the brain, but not initially considered (Persengiev et al., 2011). Several genes exhibit divergent expression patterns relative to other mammals, such as CF transmembrane conductance regulator (CFTR), RAD51 recombinase (RAD51), although they do not directly account for interspecies differences (Jiang et al., 2018). Among the 137 genes potentially associated with genetic diversity in the human lineage, some have been found to have distinct expression profiles in specific populations; e.g., glial fibrillary acidic protein (GFAP) and matrix metallopeptidase 9 (MMP9) are highly expressed in AD populations (Arendt et al., 2017; Testa et al., 2016). Actually, the evolutionary features of some of these genes have been explored. For example, interleukin 4 (IL4) mutation was caused by pathology, and the adaptive changes of COP9 signalosome subunit 5 (COPS5) were caused by environmental factors (Stobdan et al., 2017; Vitale et al., 2019). We propose that these genes collectively shape the landscape of interspecific and intraspecific genetic variation among brain-specific expressed genes. While some of these genes are involved in basic functions such as signal processing, most of the genes are related to growth and development, the nervous system, and a few genes are involved in immune and metabolic processes.

### Single-cell validation of differential expression of DEinter genes and pathways

We integrated recently published human brain organoid single-cell datasets (GSE293664, GSE302350) and macaque brain single-cell datasets (GSE319557, GSE183052) to obtain a single-cell dataset containing 131,759 cells. ssGSEA scoring of the DEinter revealed a highly significant difference in signature levels between species (Wilcoxon rank-sum test, *P*-value < 2.22e-16), with significantly higher expression in the human group (Figures 3A,B). This high expression was primarily driven by expression in excitatory neurons and non-neuronal cells (Supplementary Figure 3c). Further KEGG analysis showed that 8 out of the 13 significantly enriched pathways from the DEinter were replicated in the single-cell dataset, confirming the differential pathway expression. Notably, the Cell Adhesion Molecules and AD pathways were significantly downregulated in the human brain, and such expression patterns were affected by non-neuronal cells and Excitatory neurons, respectively (Supplementary Figures 3d,e), suggesting the evolutionary differences in cell adhesion signaling and neurodegenerative disease pathways between humans and non-human primates (Figures 3C–E).

**FIGURE 3:**
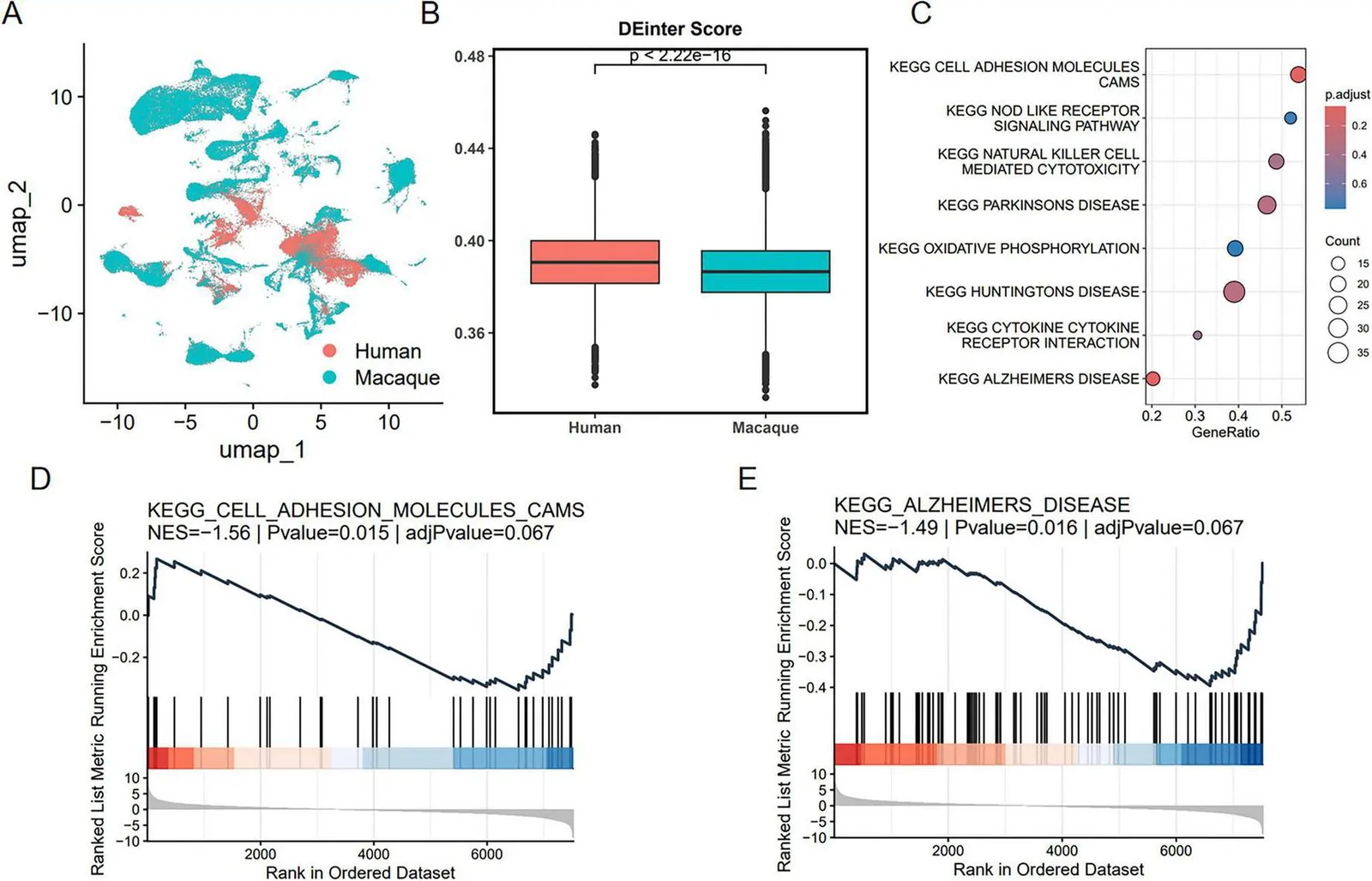
**(A)** UMAP embedding of transcriptomes, colored by species (Human: red; Macaque: blue). **(B)** Boxplot of DEinter Score. **(C)** Bubble plot of top KEGG pathways enriched in human and macaque DEGs. **(D)** GSEA plot for KEGG_CELL_ADHESION_MOLECULES_CAMS. **(E)** GSEA plot for KEGG_ALZHEIMERS_DISEASE.

## Discussion

Accumulating evidence indicates structural differences exist between human and macaque brains, with evolutionary divergence between the two species in hemispheric asymmetry of gray matter volume, anatomical connectivity, and functional connectivity within brain regions (Sun et al., 2026). Meanwhile, brain structural variations are also observed across human populations. Previous studies have identified inter-population differences in cortical thickness, gray matter volume, and cortical surface area across multiple brain regions (Tang et al., 2018). Different structures guide the development of neural circuits and ultimately may contribute to human-unique functional specializations of the central nervous system (Silbereis et al., 2016). Therefore, it is of great importance to explore the difference between human and non-human primates to recognize the phenotypic characteristics of human brain specialization at the molecular level (Preuss et al., 2004). In recent years, research in human brain evolution has increasingly focused on coding-sequence divergence, with particular emphasis on regulatory evolution, cell-type specificity, and comparative single-cell genomics. Such approaches can reveal lineage-specific chromatin remodeling, cell-type-specific transcriptional differences, and heterochronic development, among other features (Nehme et al., 2024; Zhu et al., 2018). Centering on human brain coding sequences and leveraging network-based analytical approaches, this study elucidates the genetic diversity of the human brain and offers complementary transcriptional insights into its evolution. Previous comparative transcriptomic work mainly focused on gene-expression divergence across brain cell types and brain regions between human and macaque. Building upon those expression-derived gene sets, the present study explored interspecific and intraspecific genetic diversity at the coding-sequence level for genes with brain-region or cell-type expression, as well as the genes of high evolutionary rates within PPI networks.

In this study, we performed enrichment analysis on the topological characteristics of genes in the PPI network with significant evolutionary rates. We found that the DEinter genes with lower median node degrees are functionally associated with NK cell cytotoxicity and the negative regulation of immune effector responses, and these nodes are under weaker purifying selection (Supplementary Figure 5). The evolutionary differences also encompass pathways and genes associated with neurodegenerative diseases. In addition, greedy search inferred potential genes associated with significant evolutionary rates from known genes, which have the significance of association and expansion.

Genome-wide datasets of human brain sequences with high evolutionary rates provide a convenient approach for identifying genetic diversity underlying human brain function. Significant levels of evolutionary rates of genes detected by outliers were considered for subsequent analysis. For example, lineage-specific activated genes have been demonstrated to facilitate cortical expansion (Suzuki et al., 2018). The node degree of outlier genes found based on the PPI network reflected that the genes with low node degree are under weaker purifying selection. Genes with empty nodes were not considered in the degree distribution, and evolutionary evidence has actually been reported. For example, the CD3e molecule (CD3E) and 10 other genes are thought to have been subjected to positive selection in human evolution. Defensin beta 1 (DEFB1) shows an intraspecies selection characteristic (Ohtani et al., 2011; Ortiz et al., 2009). Some interspecies empty node degrees have been studied only intraspecies. Mitochondrial coevolution may alter NADH: ubiquinone oxidoreductase subunit C2 (NDUFC2) and modulate diabetes susceptibility in specific human populations. Brain-expressed X-linked 3 (BEX3), an essential factor for nervous system development, exhibits robust signatures of positive selection between humans and macaques (Gershoni et al., 2014). Collectively, these examples confirm widespread genetic diversity within human brain specific-expressed genes, yet their cell-type-specific regulatory dynamics and developmental timing effects remain largely uncharacterized gaps that single-cell and regulatory genomics approaches are uniquely positioned to address.

Multiple pathways or biological processes are known to transmit immune and metabolic signals to the nervous system. Neurodegenerative diseases share some symptoms, and patients with AD have a higher risk of PD, which may explain the high overlap significance of neurological diseases (Hensman Moss et al., 2017; Stephenson et al., 2018). It has been proven that neurological diseases are affected by oxidative metabolism, immune inflammation, and other factors. For example, cytokines achieve regulatory purposes by affecting nerve activity and discharge response (Kenney and Ganta, 2014; Singh A. et al., 2019; Winiarska-Mieczan et al., 2020). Therefore, there was an interaction between the pathways resulting from significantly evolutionary rates of gene enrichment. Through crosstalk analysis, an interspecies KEGG pathway interaction diagram was obtained (Figure 4). The two main parts were the immune system module and the neurological disease metabolism module, with correlations existing between the two modules. There were six pathways of enrichment of dN/dS outliers when FDR was less than or equal to 0.1. Two neurological disease pathways (AD, PD), three immune system pathways, and a metabolic disease pathway were demonstrated. At FDR < 0.05, only two pathways were identified for dN/dS outliers: Measles and Cytokine-cytokine receptor interaction.

**FIGURE 4:**
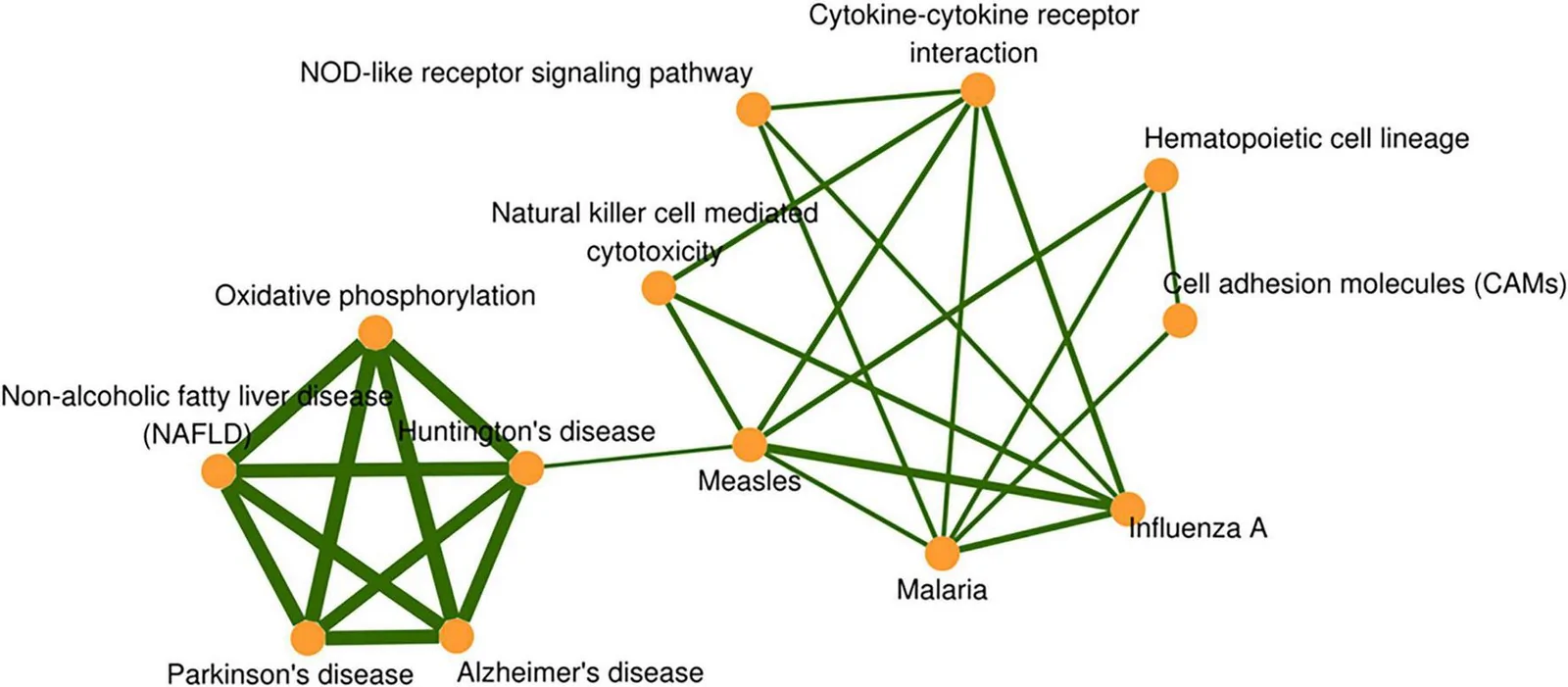
Crosstalk networks of pathways enriched in genes of DEinter. The vertices represent biological pathways, and the width of the lines is proportional to the overlap significance of pathway pairs.

Considering the integrity and potential of evolutionary rate genes, we applied greedy search to find the specific subnetworks connecting all significant evolutionary rate genes in the PPI network. Similar analyses have been reported in recent literature (Sharma et al., 2018). In the network, 23 genes (*APP*, *BAG3*, *CANX*, *CARD8*, *COPS5*, *COPS6*, *CREB3*, *DYNLL2*, *ELAVL1*, *EWSR1*, *GRB2*, *HSP90AB1*, *KRTAP10*-3, *LATS2*, *NRF1*, *PPP1CC*, *PRKACA*, *SIAH1*, *SUMO1*, *SUMO2*, *TERF1*, *TFAP2C*, *TFF1*) were shared by 97 and 137 linker genes. We speculate that these genes may shape the genetic diversity on human brain genetic diversity owing to their high node degrees. Our findings are consistent with previous reports. For example, the small ubiquitin-like modifier (SUMO) protein family has been explored the molecular mechanism through sequence alignment of multiple species. And intraspecies studies suggest that there are differences in various subtypes of SUMO (Matic et al., 2008; Staub et al., 2002). Nuclear respiratory factor 1 (NRF1) has been shown to insert amino acids in conserved regions between vertebrates, and telomeric repeat binding factor 1 (TERF1) has been proven to have intraspecific differences (Frühbeck et al., 2020; Savage et al., 2005). As expected, the shared genes with a higher degree were more evolutionarily conservative. In addition, there is evidence that changes in immune types lead to intraspecies gene differences. Recent studies have revealed that expression levels of programmed cell death 1 (PDCD1) are associated with tumor pathogenesis. Likewise, transporter 2, ATP-binding cassette subfamily B member (TAP2) exhibits disease-related nucleotide diversity, and transcription factor AP-2 gamma (TFAP2C) shows elevated expression in lung disorders (Cagliani et al., 2011; Cheng et al., 2020; Sampedro-Núñez et al., 2018). Further topological analysis of linker genes based on PPI network node degree statistics revealed that the node degrees of linker genes from DEinter exhibited statistically significant differences compared with other DEinter genes (Vuong test *P* = 0.0174), and linker genes possessed higher node degrees. The linker genes derived from DEinter showed no statistically significant difference (Supplementary Figure 4). Knockout of high-connectivity hub genes readily triggers the collapse of biological system functions; such genes show greater sequence conservation relative to DEinter genes. Nevertheless, this topological-evolutionary trend still requires further validation with expanded taxonomic sampling and experimental evidence. Subnetwork mining of potential evolutionary rate genes contributes to understanding the genetic diversity of the human brain at the gene level and provides methodological frameworks for investigating genetic diversity within the nervous system.

In this study, we identified 450 DEinter genes and 534 DEintra genes. DEinter genes with lower median node degrees exhibit functions related to NK cell cytotoxicity and negative regulation of immune effector responses, and these nodes are subject to weaker purifying selection. In particular, the greedy search revealed 97 or 137 potential genes for evolution between and within species, with previous studies providing much genetic diversity evidence. Nevertheless, this study was mainly based on the pooled datasets for human populations, with no separate calculations of pN/pS ratios and selection pressure analyses for individual subgroups; and independent calculations stratified by distinct populations will be required in future work to precisely decipher population-specific evolutionary signatures of the human brain. We note that genes with extremely few synonymous variants may yield noisy pN/pS estimates. Furthermore, our analysis was restricted to genes with non-zero cell-cluster specificity scores. This may miss evolutionarily meaningful information harbored by broadly brain-expressed genes. Future studies should incorporate both broadly expressed and cell-type-specific brain genes to provide a more complete picture of human-brain genetic diversity. The detected interspecific and intraspecific evolutionary signatures only provide indirect brain-related evidence. Owing to unavailable matched human-macaque multi-tissue data and widespread gene pleiotropy, interference from peripheral tissue selection cannot be excluded. Further functional validation is required to verify brain-specific evolutionary adaptation. We also recognize that rigorous testing of whether the magnitude of the node degree-evolutionary rate correlation is stronger in brain would require matched multi-tissue expression datasets and systematic classification of tissue-specific genes. We did not identify evolutionary outliers after accounting for CDS length, GC content, and synonymous substitution saturation. Another important limitation stems from our exploratory single-cell level analyses. Brain organoids show transcriptomic differences relative to adult *in vivo* brain tissue. For this reason, these results should be regarded only as exploratory observations rather than rigorous cross-species validation. Future studies will require well-matched single-cell datasets of adult primate brains to perform more reliable interspecies comparisons.

Compared with previous aggregated pathway-level analyses, the present study shifts the analytical perspective and dissects the genetic diversity of human brain genes starting from outlier genes with high evolutionary rates. We demonstrate that evolutionary signals in pathways related to neurological diseases, immunity, and metabolism are mainly driven by a small subset of extreme dN/dS and pN/pS outlier genes within those pathways. Using the PPI network combined with the Steiner minimal tree algorithm, we find that these evolutionary outlier genes mostly occupy peripheral positions in the PPI network and are subject to relatively weaker purifying selection. We also identify a set of underappreciated linker genes. Meanwhile, this study addresses the confounding effects of covariates including CDS length, GC content, and dS saturation on the evolutionary metrics dN/dS and pN/pS. By leveraging cross-species single-cell transcriptomic data, we further refine the bulk-level evolutionary differences observed in pathways and map them onto specific brain cell subpopulations. Taken together, from multiple dimensions including individual gene dissection, topological features of PPI networks, confounding factor evaluation, and single-cell subtype validation, this study expands and deepens our understanding of the genetic diversity of coding sequences in the human brain.

In summary, by systematically exploring genes with evolutionary rates in the human brain from interspecific and intraspecific perspectives, this study reveals the genetic diversity of human brain specific-expressed genes and offers complementary insights into human brain evolution at the transcriptional level.

## Data Availability

The original contributions presented in this study are included in this article/Supplementary material, further inquiries can be directed to the corresponding authors.

## Footnotes

1
https://doi.org/10.1038/354116a0

2
http://www.ensembl.org/

3
https://www.internationalgenome.org/
