# Supplementary material for: Network-based analysis reveals genetic diversity of human-brain expressed genes with high evolutionary rates
Source: Front Neurosci. 2026 Oct 20;20:1866277. doi: 10.3389/fnins.2026.1866277 (PMC13727637; doi:10.3389/fnins.2026.1866277)
Supplement: Supplementary file 1 [file Supplementary_file_1.pdf]

Figure S1

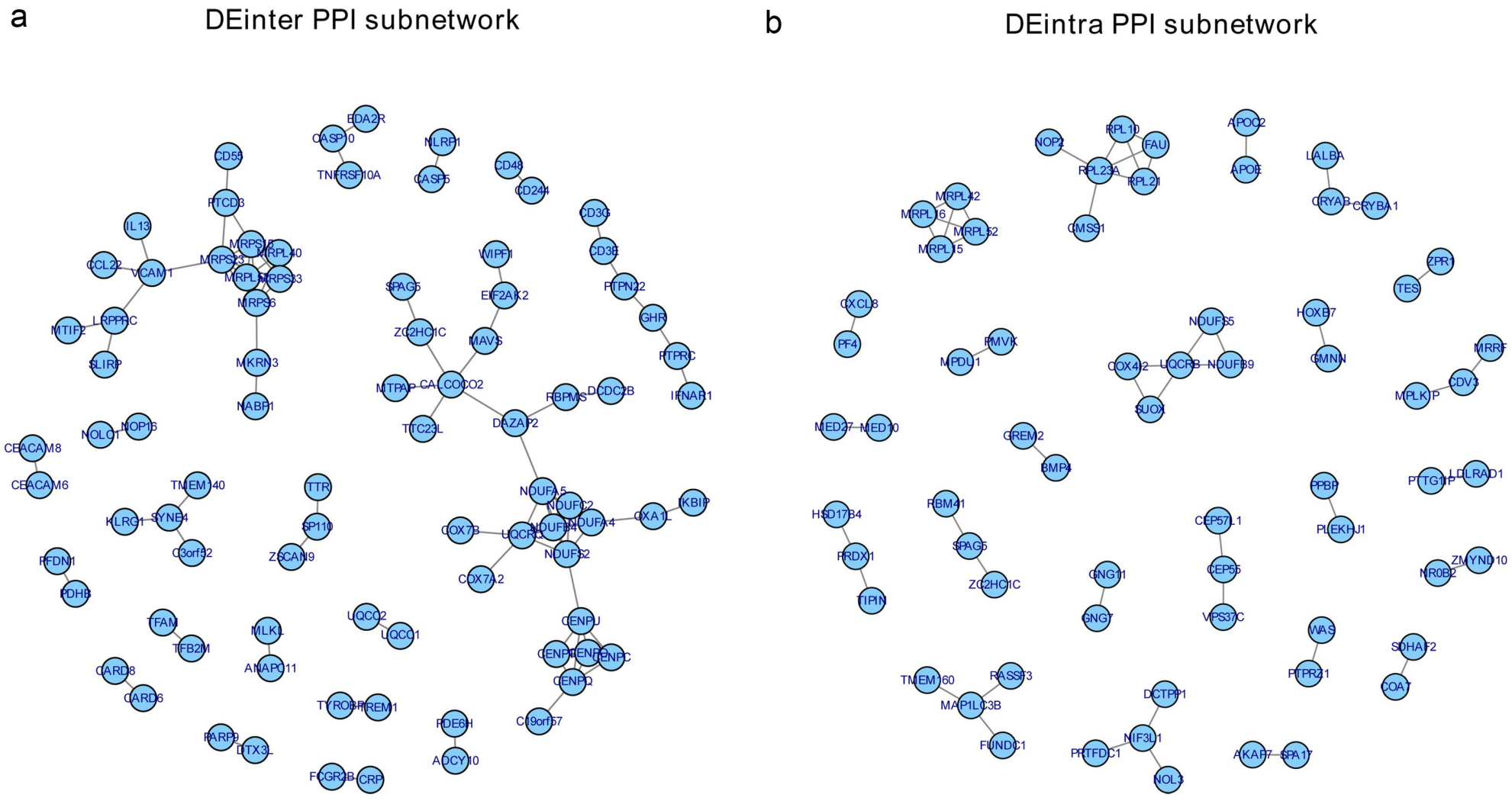

Figure S1. PPI subnets for DEinter (a) and DEintra (b), with statistical comparison of betweenness centrality between the two modules.

Figure S2

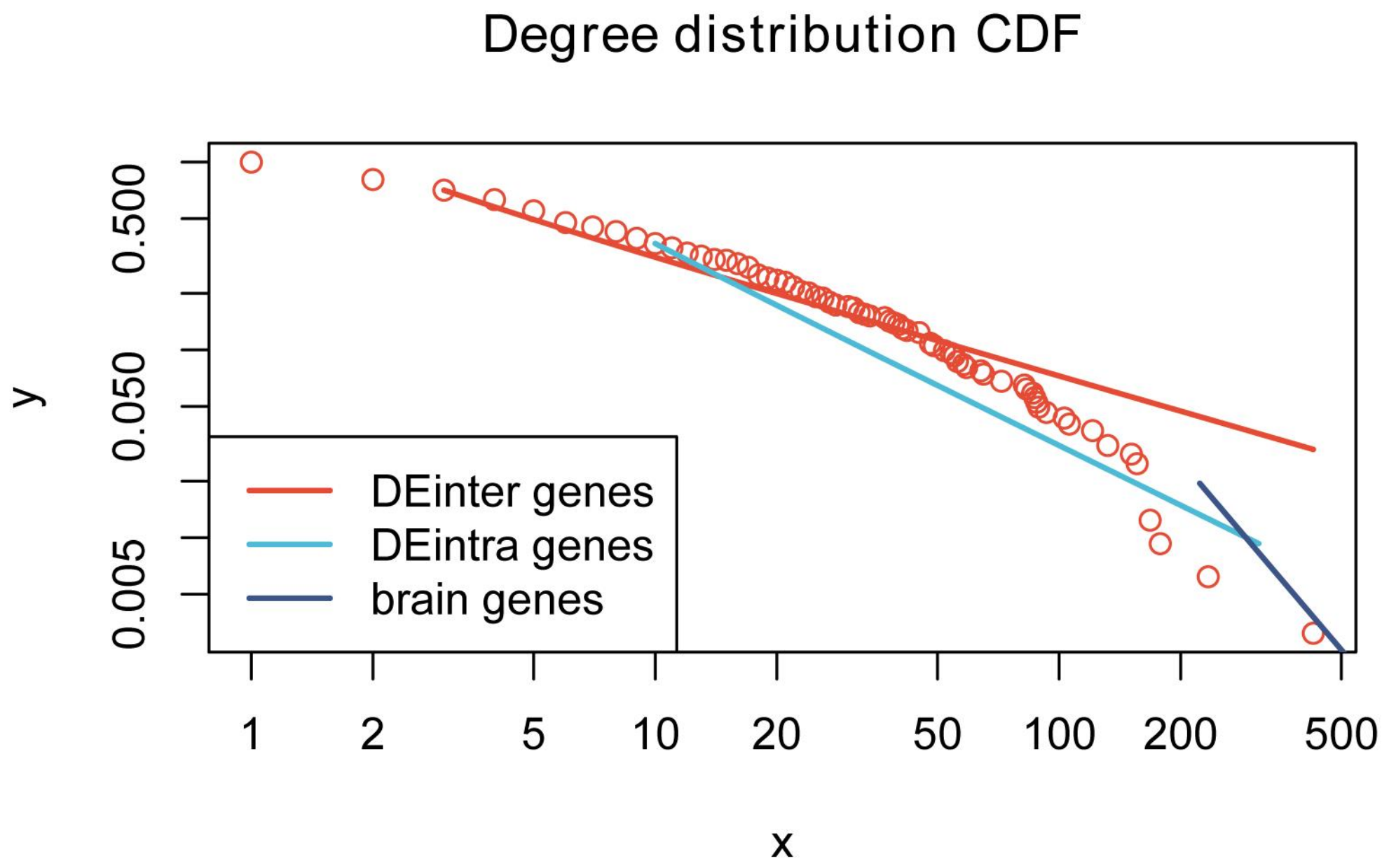

Figure S2. The CDF curves of degree distributions for DEinter, DEintra, and brain genes. Red circles show empirical data of DEinter genes, solid colored lines denote MLE power-law tail fits for each group (red is DEinter genes, blue is DEintra genes, deep blue is brain genes).

Figure S3

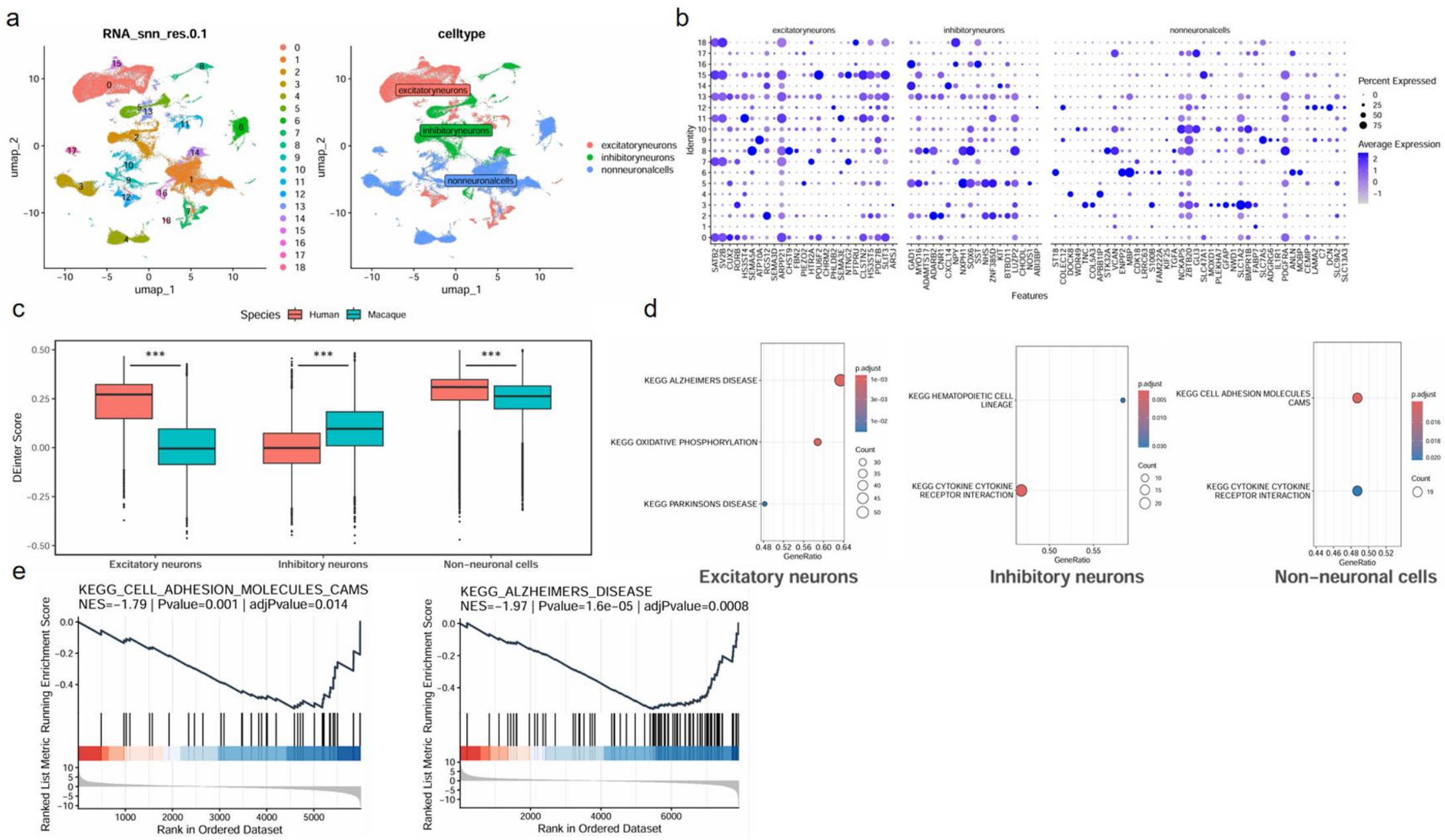

Figure S3. (a) UMAP visualization of unsupervised clusters (left) and annotated cell types (right). (b) Dot plot of marker gene expression. (c) Boxplots of DEinter scores comparing human and macaque transcriptomes, with *** indicating statistical significance (P value < 0.001). (d) Bubble plot of top KEGG pathways for cell-type-specific differential genes. (e) GSEA plot for KEGG_CELL_ADHESION_MOLECULES_CAMS (left) and KEGG_ALZHEIMERS_DISEASE (right).

Figure S4

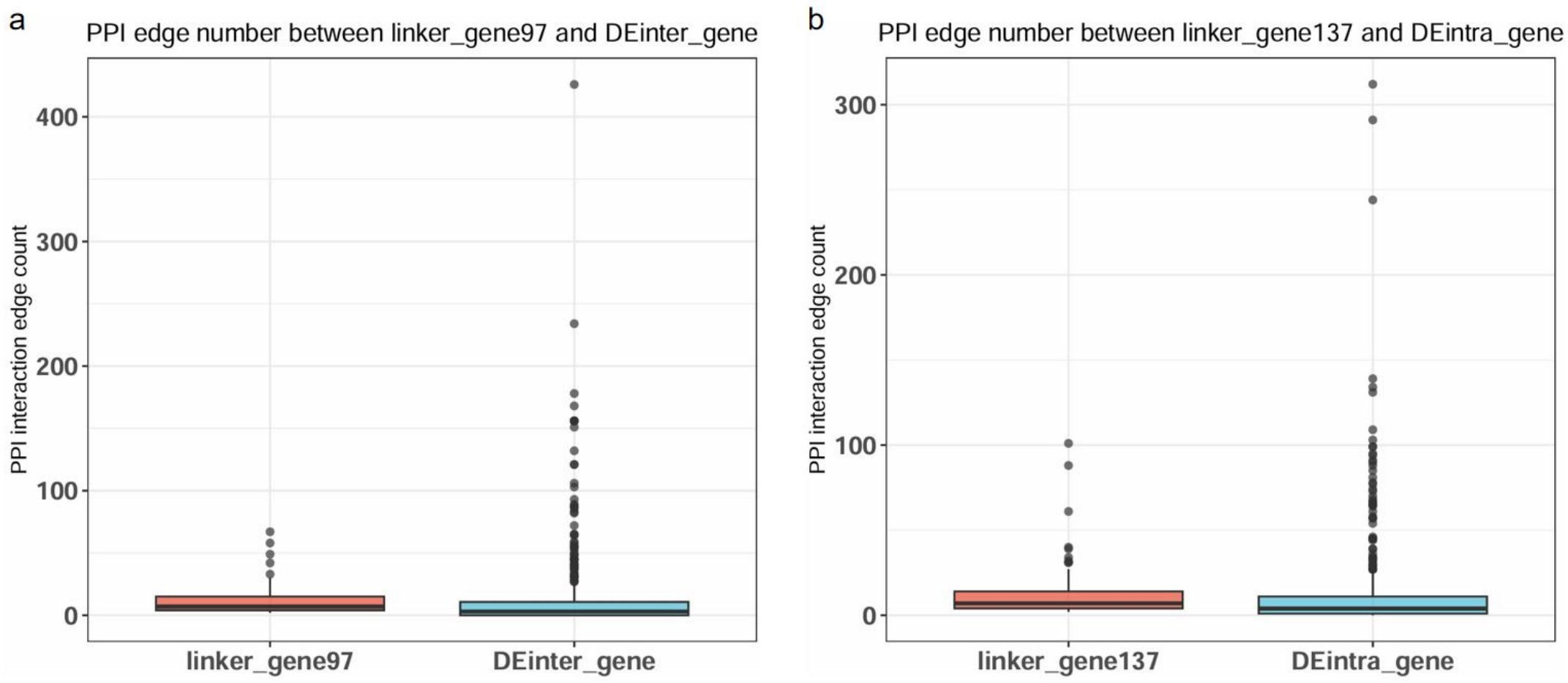

Figure S4. PPI interaction edge count distributions. (a) linker_gene97 vs DEinter genes; (b) linker_gene137 vs DEintra genes. DEinter and DEintra genes possess higher median PPI interaction numbers and more high-edge outliers than their respective linker gene sets.

Figure S5

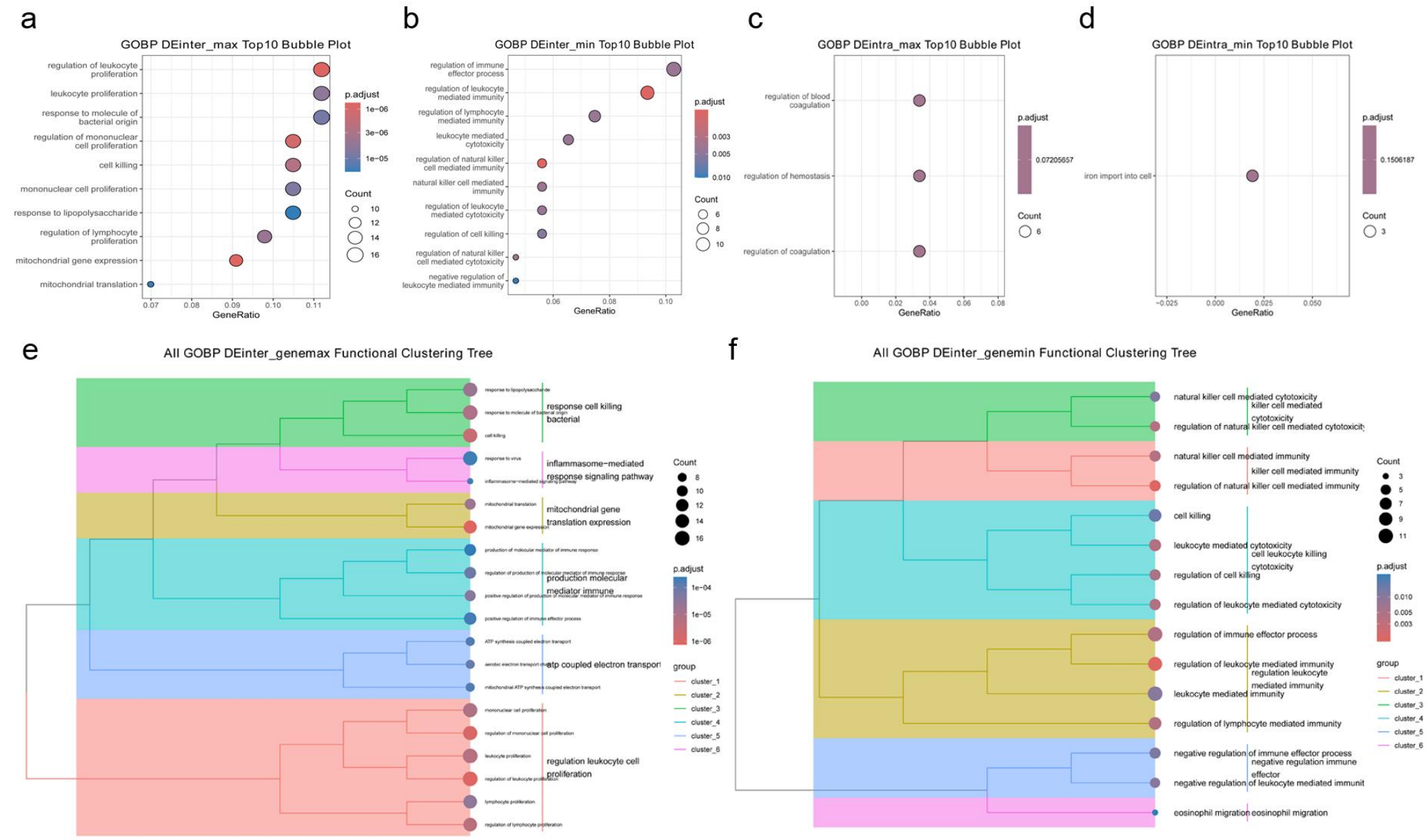

Figure S5. GOBP enrichment and functional clustering. The dotplot of the top 10 GOBP for DEinter_max (a) and DEinter_min (b); The dotplot of GOBP for DEintra_max (c) and DEintra_min (d). Functional clustering trees of all enriched GOBP from DEinter_genemax (e) and DEinter_genemin (f), with six color-coded functional clusters, dot size for gene count, and color gradient denotes adjusted P-value.

Figure S6

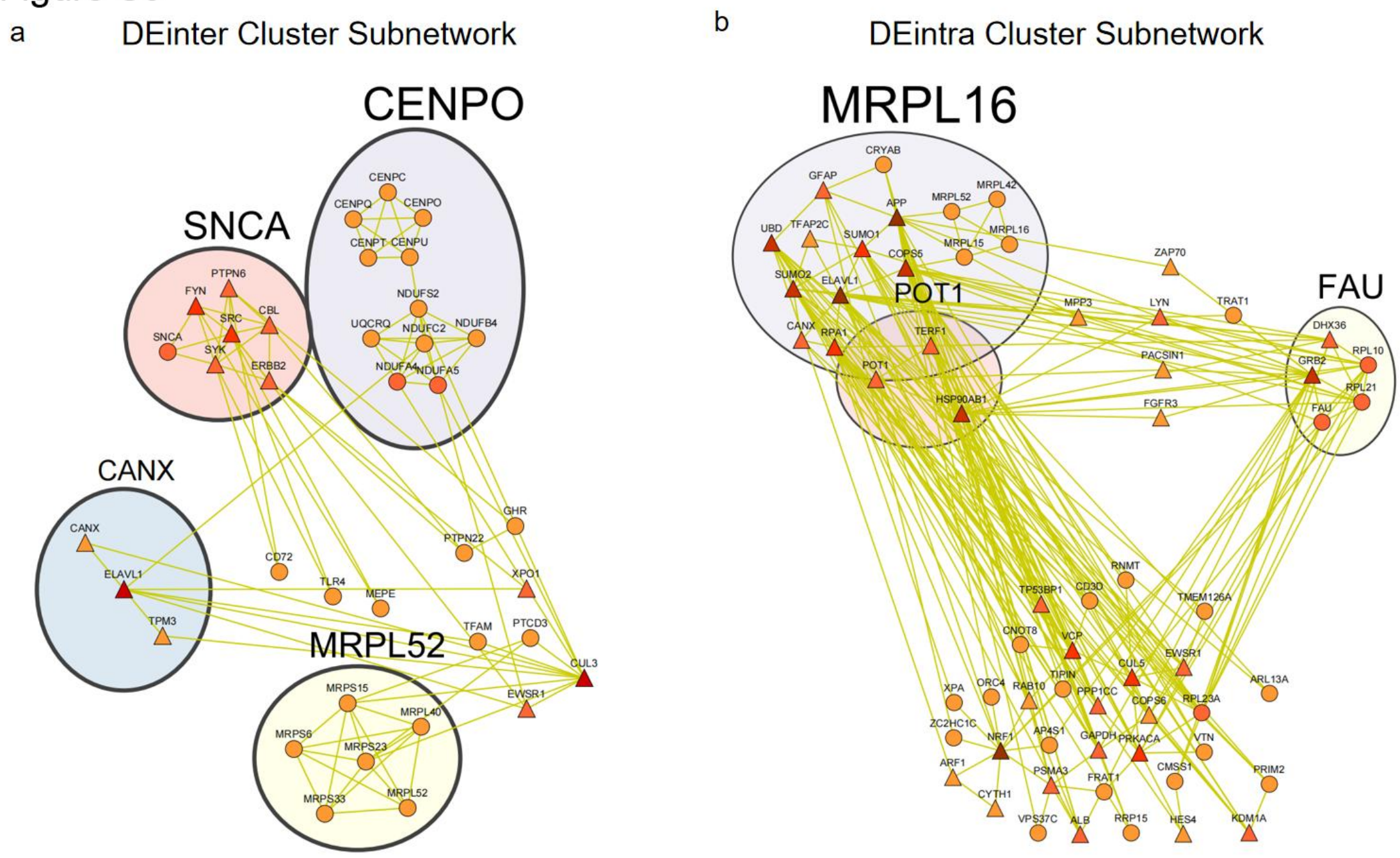

Figure S6. Subnetwork of gene-specific clusters for standing variation. (a) The DEinter cluster subnetwork , which consists of 37 nodes and 88 edges. (b) The DEintra cluster subnetwork , which consists of 62 nodes and 201 edges.

Figure S7

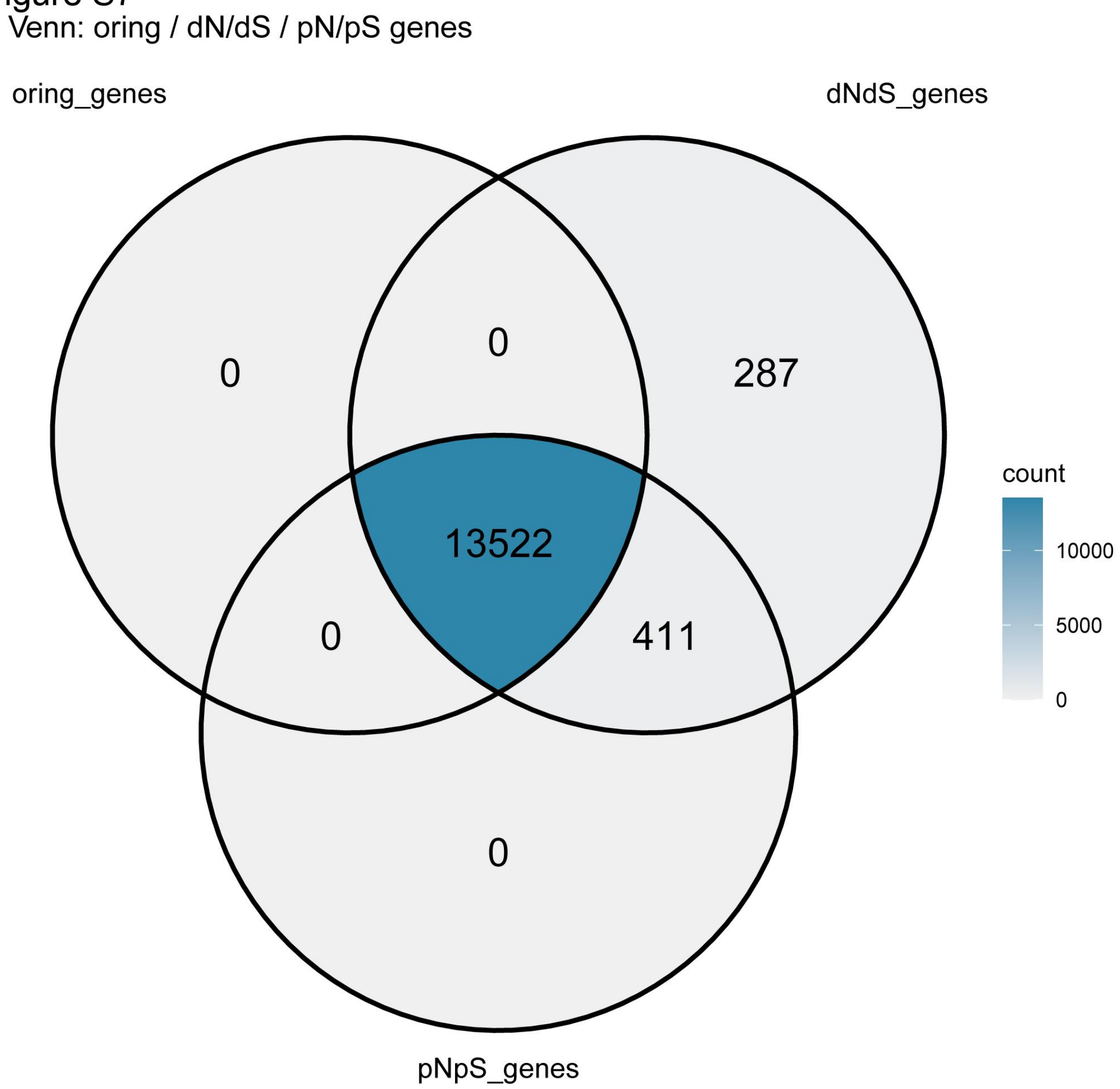

Figure S7. Venn diagram showing overlaps of oring_genes, dNdS_genes and pNpS_genes. The triple-overlapping region (blue) represents 13522 shared genes with valid non-zero oring, dN/dS and pN/pS values. Values within each partition correspond to gene numbers.

Figure S8

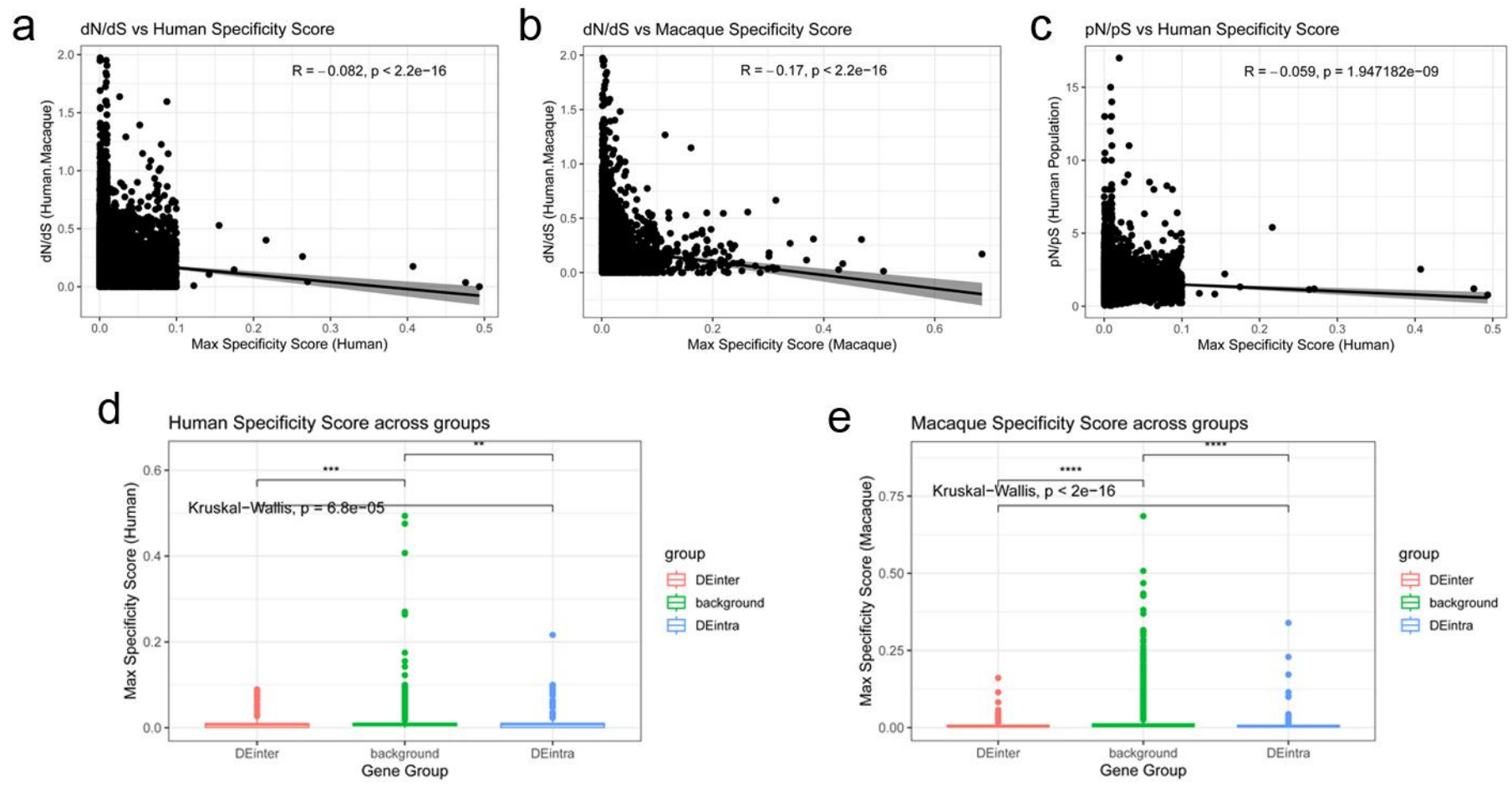

Figure S8. Spearman's rank correlation coefficient analyses between dN/dS, pN/pS and maximal cell-type specificity scores. (a) Correlation between dN/dS and human specificity score. (b) Correlation between dN/dS and macaque specificity score. (c) Correlation between pN/pS and human specificity score. Each dot represents an individual gene. The black line denotes the regression trend line, and the gray shaded area indicates the 95% confidence interval. Boxplots show the distribution of max specificity score across the DEinter, DEintra, and background gene groups. (d) Human specificity score across groups (e) Macaque specificity score across groups. ** $P < 0.01$; *** $P < 0.001$, comparisons with $P \geq 0.05$ are unmarked.

Figure S9

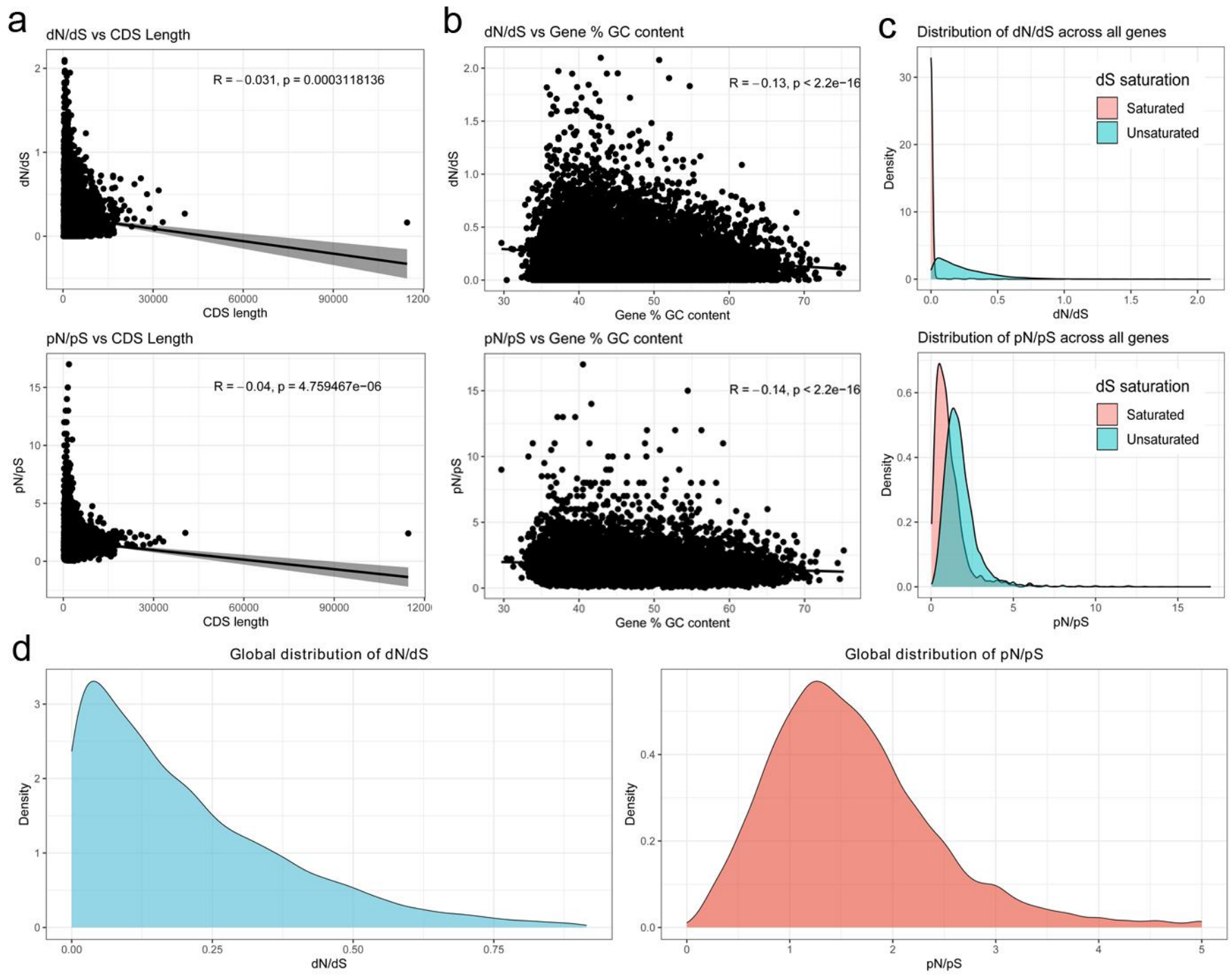

Figure S9. Correlations of dN/dS and pN/pS with CDS length and gene GC content, and global distributions of dN/dS and pN/pS across all genes. (a) Correlation between dN/dS and CDS length (top), and correlation between pN/pS and CDS length (bottom). (b) Correlation between dN/dS and gene % GC content (top), and correlation between pN/pS and gene % GC content (bottom). Black lines denote regression trend lines, and gray shaded areas indicate 95% confidence intervals. (c) Density distributions of dN/dS (top) and pN/pS (bottom) stratified by dS saturation status (saturated, red; unsaturated, cyan). (d) Global density distributions of dN/dS (left) and pN/pS (right) for all genes.
